# Magazines and Exchanges

**Published:** 1898-10

**Authors:** 


### *MAGAZINES AND EXCHANGES.*

---

*The Psychiater* is a new publication which has just issued its first number. It is published and edited at the Hospital Illinois by Wm. G. Stearns, M.D., Superintendent of the Illinois Eastern Hospital for Insane, and other members of the medical staff. The first issue is well gotten up and presents some most excellent reading-matter. We welcome it among our exchanges. It will be issued four times a year.

---

WE note among our exchanges the appearance of the *Crónica Médico-Quirurgica de la Habana*, which we had not seen for many months. The cessation of hostilities and the resuming of business in Havana has permitted again the publication of this journal. Its editor, Dr. J. Santos Fernandez, is a man of great ability, and we welcome again to our list the publication of our confrère, which while it was not dead, was yet sleeping. The issue is entirely taken up with the life of Dr. D. Domingo Madan, of Cuba, a man evidently much beloved and of marked ability. We print elsewhere this life of the man, translated from the Spanish by Mr. B. J. Word, of this city.

---

MR. W. B. SAUNDERS, the medical publisher, is giving to the medical profession some excellent publications with which to stock his library. The hand-atlases are gems in every respect. Mr. Saunders is making preparations for the production of these atlases on a larger scale, since the sales have been up to this time so phenomenal.

The following are some of the publications which have already been issued from this house, or are in preparation for an early issue :

1. Da Costa's Modern Surgery.
2. McFarland's Pathogenic Bacteria.
3. An American Text-book of Diseases of Children.
4. An American Text-book of Gynecology.
5. Vierordt's Medical Diagnosis.
6. Griffith's Care of the Baby.
7. Butler's Materia Medica and Therapeutics.
8. Stengel's Text-book of Pathology.
9. Hirst's Text-book of Obstetrics.
10. American Text-book of Diseases of Eye, Ear, Nose and Throat.

